# Event-related potentials when identifying or color-naming threatening schematic stimuli in spider phobic and non-phobic individuals

**DOI:** 10.1186/1471-244X-6-38

**Published:** 2006-09-18

**Authors:** Iris-Tatjana Kolassa, Frauke Musial, Stephan Kolassa, Wolfgang HR Miltner

**Affiliations:** 1 Biological and Clinical Psychology, Friedrich Schiller University Jena, Germany; 2 Clinical Psychology & Neuropsychology, University of Konstanz, P.O. Box 5560, D25, 78457 Konstanz, Germany

## Abstract

**Background:**

Previous studies revealed increased parietal late positive potentials (LPPs) in response to spider pictures in spider phobic individuals. This study searched for basic features of fear-relevant stimuli by investigating whether schematic spider images are sufficient to evoke differential behavioral as well as differential early and late ERP responses in spider phobic, social phobic (as a clinical control group), and non-phobic control participants.

**Methods:**

Behavioral and electrophysiological correlates of the processing of schematic spider and flower images were investigated while participants performed a color (emotional Stroop) and an object identification task. Stimuli were schematic pictures of spiders and flowers matched with respect to constituting visual elements.

**Results:**

Consistent with previous studies using photographic spider pictures, spider phobic persons showed enhanced LPPs when identifying schematic spiders compared to schematic flowers. In addition, spider phobic individuals showed generally faster responses than the control groups. This effect was interpreted as evidence for an increased general behavioral hypervigilance in this anxiety disorder group. Furthermore, both phobic groups showed enhanced P100 amplitudes compared to controls, which was interpreted as evidence for an increased (cortical) hypervigilance for incoming stimuli in phobic patients in general. Finally, all groups showed faster identification of and larger N170 amplitudes in response to schematic spider than flower pictures. This may reflect either a general advantage for fear-relevant compared to neutral stimuli, or might be due to a higher level of expertise in processing schematic spiders as compared to the more artificially looking flower stimuli.

**Conclusion:**

Results suggest that schematic spiders are sufficient to prompt differential responses in spider-fearful and spider-non-fearful persons in late ERP components. Early ERP components, on the other hand, seem to be modified by anxiety status per se, which is consistent with recent theories on general hypervigilance in the anxiety disorder spectrum.

## Background

Spiders are genuinely feared stimuli for individuals with spider phobia, but they are also considered fear-relevant (ancestral) stimuli for humans in general, and it has been hypothesized that such stimuli are detected and processed preferentially to other stimuli [1-4]. In support of this hypothesis, Öhman, Flykt, and Esteves [5] reported faster detection of fear-relevant stimuli among neutral stimuli than vice versa in a visual search task (however, see [6] for a critical comment on the study). Furthermore, phobic persons detected feared stimuli even faster than fear-relevant stimuli they did not fear [5]. Thus, it has been suggested that phobic individuals are characterized by an attentional bias towards their feared object, i.e., phobics' attention is drawn involuntarily and automatically to the feared object, and they process it with high selectivity and priority ([7-9], for an overview see [9]).

However, in a visual search paradigm Miltner et al. [6] observed no threat advantage in spider phobic and control persons for spiders when participants' task was to search for a spider target stimulus among a crowd of neutral stimuli displayed in a 4 × 4 grid pattern search array. Instead, they found delayed responses in spider phobic individuals to a neutral target (mushroom) in a crowd of neutral objects (flowers) in the presence of a spider distractor. In the same series of studies, by measuring eye movements they observed that phobic individuals first moved their eyes toward the spider distractor before focusing on the neutral target, delaying phobics' target detection times. These findings might suggest that an attentional bias is present in phobic persons only if the feared stimulus is not the focus of attention (cf. also [10]).

One paradigm that allows the investigation of the processing of threatening stimuli when the threatening information itself is not task-relevant is the emotional Stroop paradigm. The emotional Stroop paradigm is a modified version of the original Stroop task [11] and is commonly used to assess attentional biases in anxiety disorders or high trait anxiety (for an overview see [9]). In this paradigm, the difference in color-naming latencies between emotionally relevant and emotionally neutral words or pictures is measured. The phenomenon that anxious persons show prolonged response latencies when color-naming feared stimuli has been called emotional Stroop interference (see [9]). Numerous studies have reported emotional interference in animal phobic individuals ([12-17]; but see [18]). The emotional Stroop paradigm can be regarded as an *implicit *task, i.e. the emotional content of the stimulus is not the focus of attention, as compared to an *explicit *task in which subjects explicitly identify the emotional content of the stimulus. It has been shown that task conditions affect brain activation to emotional stimuli [10,19-21], and thus implicit and explicit tasks should be compared when investigating the processing of fear-related stimuli.

Although spiders are commonly regarded as fear-relevant stimuli, it remains largely unclear which perceptual properties make a spider fear-relevant. It has been postulated that specific feature detectors with high sensitivity to elementary threat features exist, "programmed" either genetically or by conditioning (cf. [1,22]). These detectors screen incoming information for specific threat cues (e.g. high intensity or biologically prepared stimulus characteristics). As soon as a specific threat feature has been detected, the arousal system is activated still preattentively, and the stimulus is selected for preferential treatment by succeeding stages of stimulus elaboration [1]. However, as Öhman et al. ([5], p. 475) remark, "such elementary threat features [. . .] still remain to be specified".

This study searched for basic features of fear-relevant stimuli by investigating whether schematic spider pictures are sufficient to evoke differential responses in spider phobic and spider-non-phobic individuals, in order to provide first insights regarding the question which properties constitute the fear-relevance of a spider. The advantages of schematic stimuli are obvious: schematic stimuli reduce the depicted object to its essential features and are therefore simple and unequivocal. Schematic stimuli show less variance: in the case of spider stimuli there is no confoundation with spider species, hairiness, size, or camera angle. Finally, it is easier to design a control stimulus matched with respect to color, size, and spatial frequency: if one shifts the angles of the legs of a schematic spider image a schematic flower picture results.

Preceding studies using real spider pictures reported that spider phobic individuals exhibited larger parietal late positive potentials (LPPs) in response to and a faster identification of spiders than birds or flowers as well as larger LPPs and faster reaction times in response to spider pictures than controls and social phobic persons ([18]; compare also [23]). Spider-non-fearful persons also showed a trend towards larger LPPs for spiders than flowers. The enhanced LPPs in response to spiders in the spider phobic group in particular were interpreted as a correlate of increased perceptual processing of these stimuli. ERP studies consistently found larger LPPs for emotional, both pleasant and unpleasant, compared to neutral stimuli [24-35].

Furthermore, if the attentional bias observed in persons with specific phobia is caused by abnormalities in the early visual processing stream, ERP studies should offer evidence for such abnormal processes in early visual components such as the occipital P100 and the posterior occipito-temporal N170 component – in support of this view, Straube, Mentzel, and Miltner [36] observed increased activation of extrastriate visual cortex in social phobic persons compared to non-phobic controls when processing faces in an fMRI study. The P100 is known to be an attention-sensitive component (for a review see [37]). The N170, although generally assumed to indicate face processing mechanisms [38,39], also seems to be modulated by perceptual expertise in discriminating objects [40-42]. For example, Tanaka and Curran [42] reported larger N170 amplitudes when experts categorized objects in their domain of expertise relative to objects outside their domain of expertise. Because spider phobics might be experts in the processing of spiders, they could thus be expected to show larger N170 amplitudes for spiders than neutral objects.

Following the experimental design of Kolassa et al. [18], this study used a modified emotional Stroop task (implicit task) and an object identification task (explicit task): in the former participants identified the color of red or blue schematic spiders or flowers, in the latter they classified the stimulus category. Participants were individuals with spider phobia, social phobic persons as an additional clinical control group, and non-phobic controls. In the color identification task, we expected longer response latencies for the color identification of spiders compared to flowers in spider phobics, i.e. emotional Stroop interference. Furthermore, in the object identification task, a faster identification of spiders in general and in particular by spider phobics was expected. Regarding ERP data, larger LPPs in response to spiders in general and in spider phobic individuals in particular were expected. Furthermore, we expected spider phobic participants to respond to spiders with larger early visual ERPs, i.e., P100 and N170 components, reflecting attentional bias and expertise effects, respectively.

## Methods

### Pilot Study

In a pilot study, 43 students of the University of Jena rated schematic spider and flower pictures as to their affective valence and arousal with the Self-Assessment Manikin Scale (SAM; [43]). Fourteen of these participants were diagnosed with spider phobia (5 male, 9 female; mean age *M *= 22.3 years, *SD *= 3.8), 12 with social phobia (7 male, 5 female; mean age *M *= 26.1 years, *SD *= 3.9), and 17 were healthy controls (7 male, 10 female; mean age *M *= 23.1 years, *SD *= 3.7). Participants in the pilot study were recruited, screened and diagnosed by the same procedure as participants in the main experiment.

ANOVAs revealed significantly more unpleasant, *F*(1,40) = 50.39, *p *< .0001, η_p_^2 ^= .56, and arousing, *F*(1,40) = 36.71, *p *< .0001, η_p_^2 ^= .48, ratings for pictures displaying schematic spiders than schematic flowers (see Table 1). Furthermore, main effects of Group for valence, *F*(2,40) = 5.50, *p *= .008, η_p_^2 ^= .22, and arousal ratings, *F*(2,40) = 8.08, *p *= .001, η_p_^2 ^= .29, were observed. As expected, individuals with spider phobia rated schematic spider pictures as significantly more unpleasant, interaction of Group × Object, *F*(2,40) = 7.91, *p *= .001, η_p_^2 ^= 28, and arousing, *F*(2,40) = 11.46, *p *= .0001, η_p_^2 ^= .36, than controls and persons with social phobia. Thus, the pictures used in this study were suitable to elicit specific responses in each group.

**Table 1:** Valence and arousal ratings

	Controls		Spider Phobics		Social Phobics	
	
Rating	*M*	*SD*	*M*	*SD*	*M*	*SD*
Valence						
Spider	5.46	1.65	3.30	1.52	4.59	0.69
Flower	6.05	1.45	5.29	1.27	5.39	0.77

Arousal						
Spider	2.74	1.42	5.30	1.63	3.27	1.31
Flower	2.24	1.10	3.28	1.25	2.93	1.38

Mean (*M*) valence and arousal ratings and standard deviations (*SD*) for schematic spider and flower stimuli for each group. The SAM scale [23, 43, 79] ranges from 1 to 9, with 1 = highly unpleasant/low arousing and 9 = highly pleasant/highly arousing.

### Participants

Fifty-six individuals (mean age 23 years, *SD *= 3.5, age range 19–32 yrs) participated in the study: 18 spider phobic individuals (9 male, 9 female), 19 social phobic patients (10 male, 9 female), and 19 controls (10 male, 9 female). Groups did not differ in mean age. Fifty-three participants were right-handed and 3 left-handed as measured by the Edinburgh handedness questionnaire [44]. All participants were students of the University of Jena recruited by newspaper advertisement, on campus bulletin boards and in lectures. All procedures were approved by the ethics committee of the University of Jena. Participants provided informed consent and were paid 6 € an hour for their participation. As additional compensation participants with spider phobia were offered a one-day spider phobia therapy [45] and individuals with social phobia received a 10-session group training of social skills after the experiment [46].

Prior to the experiment proper, participants were screened with the Structured Clinical Interview for DSM-IV (SCID-I; [47]). Spider phobic and social phobic individuals were included in the study if they fulfilled DSM-IV criteria [48] of spider phobia and social phobia respectively, but did not suffer from any other current or previous disorders according to DSM-IV. Controls were accepted for participation if they had no current or past mental disorders according to DSM-IV. All participants were free of any psychotropic medication. Prior to the experiment, all participants completed German versions of the Spider Questionnaire (SPQ; [49]), the Social Phobia and Anxiety Inventory (SPAI; [50]), the Beck Depression Inventory (BDI; [51]), and the Trait Anxiety Questionnaire of the State-Trait Anxiety Inventory (STAI; [52,53]). See Table 2 for questionnaire values.

**Table 2:** Questionnaire values

	Control Group		Spider Phobics		Social Phobics		
		
Questionnaire	*M*	*SD*	*M*	*SD*	*M*	*SD*	One-way ANOVA
SPQ	2.47	1.78	20.61	2.66	2.58	1.95	*F*(2,53) = 430.97, *p *< .001*‡
SPAI	33.53	16.89	44.41	15.51	126.81	18.17	*F*(2,53) = 171.72, *p *< .001^†^‡
BDI	2.68	2.71	4.94	4.76	9.42	7.09	*F*(2,53) = 8.52, *p *= .001^†^‡
STAI-T	30.79	5.92	33.50	8.05	50.47	6.61	*F*(2,53) = 45.27, *p *< .001*‡

Means (*M*) and standard deviations (*SD*) for each group. The German scores of the Social Phobia and Anxiety Inventory (SPAI) were transformed into the original scores [80]. STAI-T, State-Trait Anxiety Inventory – Trait Version; BDI, Beck Depression Inventory; SPQ, Spider Questionnaire.

* Control group differs from spider phobics (*p *< .005). ^† ^Control group differs from social phobics (*p *< .05). ‡ Spider phobics differ from social phobics (*p *< .05)

Social phobic individuals scored on average higher on the BDI than controls and individuals with spider phobia, but the scores were not in the clinically significant range, *M *= 9.42, *SD *= 7.09. The high comorbidity of social phobia with depression is well established [54-56].

### Paradigm

**Figure 1:**
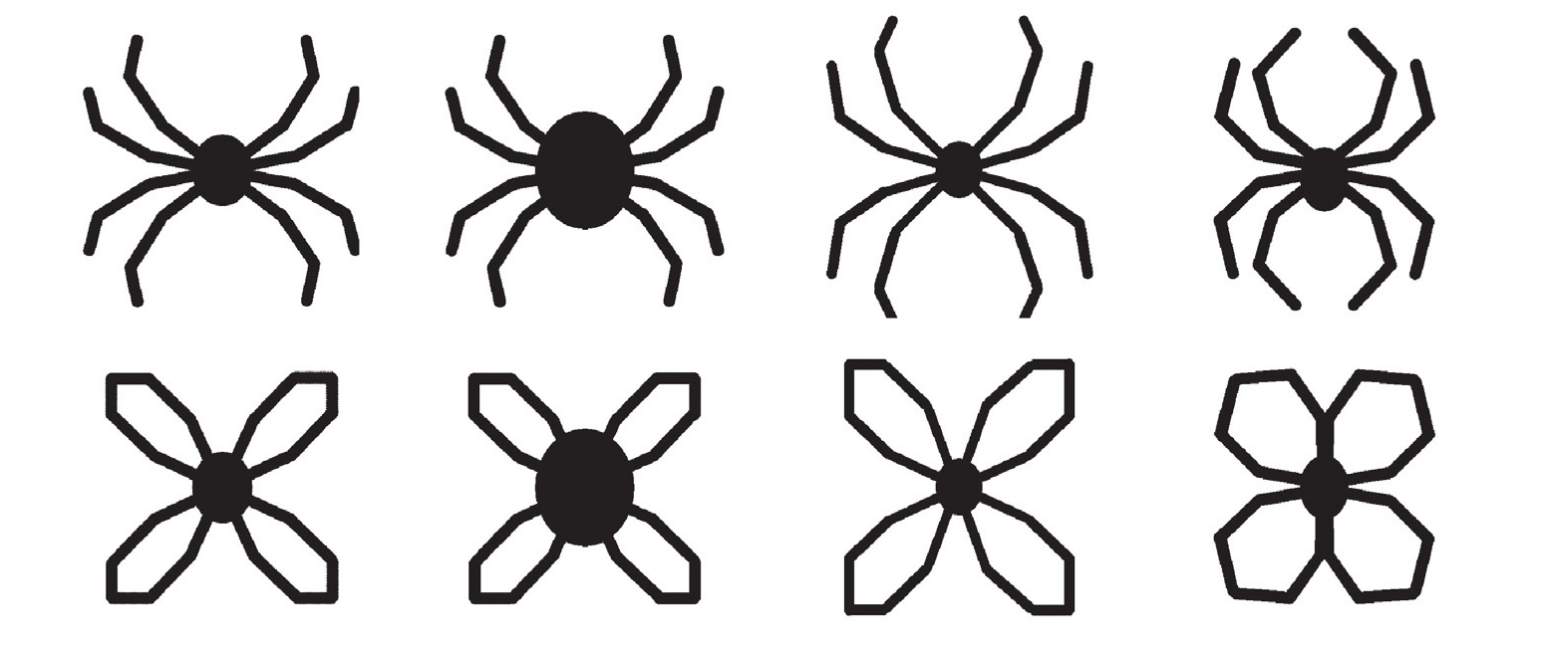
**Stimuli**. Schematic flower and spider stimuli. In the actual experiment stimuli were colored red or blue.

The experiment consisted of two blocks, each preceded by a training phase. In each block, 60 pictures of schematic spiders and flowers (30 of each) were presented for 1 s with a variable interstimulus interval of 2–3 s. Of each category 15 stimuli were colored red and the other 15 blue. Four different types of schematic flower and spider pictures were used (see Figure 1): flower stimuli varied with regard to the size of the flower's interior and the angularity of the outline petals; spider pictures differed in body size and angularity of spider legs (similar stimuli were also used by Vuilleumier and Schwartz [57]). Flowers differed from spiders only insofar as four legs of the spiders were reflected about a diagonal axis.

In one block participants' task was to identify the color (blue or red) of the stimulus, whereas in the other block they were instructed to categorize the object (spider or flower). Answers were given by pressing one of two buttons on a button box with the index finger of the dominant hand while EEG was recorded. Participants were asked to respond as quickly and as accurately as possible. The presentation order of the two blocks as well as the assignment of buttons was counterbalanced across participants. The order of the stimuli in each block was pseudorandomized: no more than 4 times did the same color and no more than 4 times did the same object category occur in a row. The occurrence of identical objects of the same color was allowed only 2 times in a row.

### Assessment and Analysis of EEG

During the testing session participants sat in a comfortable chair in a sound-attenuated room. Stimuli were presented on a 20 inch Sony monitor (resolution 800 × 600) placed 1.1 m in front of the subject. EEG was recorded with a 62-electrode montage according to the international 10-10 system [58] with additional non-standard electrodes (AF1, AF2, PO1, PO3) at frontal and occipital sites spaced equally between the standard electrodes. Cz served as a reference electrode. Electrode impedances were kept below 5 kΩ. Vertical and horizontal electrooculograms (VEOG and HEOG) were measured for off-line correction of eye movements and blink artifacts. EEG data were recorded continuously in AC mode at a rate of 500 Hz.

The EEG raw data were filtered (low pass = 30 Hz, 24 dB/oct; high pass = 0.1 Hz, 24 dB/oct), segmented (200 ms pre- to 900 ms poststimulus), corrected for blinks [59], and screened for artifacts with the software Brain Vision Analyzer 1.04 (Brain Products GmbH, Germany). Trials containing artifacts (amplitude deviations of ± 150 μV) were rejected. Altogether 2.8% of trials were excluded due to artifacts (3.0% Identify Color Spiders; 2.5% Identify Object Spiders; 3.1% Identify Color Flowers; 2.7% Identify Object Flowers). Artifact-free EEG epochs were averaged for each subject, condition and electrode. All epochs were aligned to the prestimulus baseline from -200 to 0 ms and rereferenced to an average reference.

Time intervals chosen for peak detection were based on visual inspection of the data and grand means and agreed well with those suggested by a temporal principal component analysis (PCA). Using varimax rotation, 9 components were extracted from ERPs. Factor 2 explained 16.2% of total variance and showed a broad parietal distribution with a maximum at Pz. This factor peaked at 330 ms and showed a turning point at about 370 ms after which a second prolonged positivity followed. Subjects loading high on this factor showed a pronounced positivity in this latency range in the ERP. Factor 3 explained 6.4% of total variance, was maximal on electrodes P7 and P8, and peaked at 150 ms. Subjects loading high on this factor showed a negative component on P7 and P8 electrodes in this latency range. Factor 4 explained 4.2% of total variance and had a pronounced occipital maximum at 80 ms. Subjects loading high on this component showed a pronounced positivity in this latency range. Because the other factors are of no relevance in this context, they are not further discussed here.

Factor peak-latency and topography characteristics thus associate Factor 2 with the late parietal potentials (LPPs), Factor 3 with the wave labeled N170 and Factor 4 with P100. Since it is well known that multiple positive components over parietal areas are frequently observed in response to emotional stimuli (e.g., [18,26,30]), and since it is possible that the PCA was not able to disentangle such multiple components due to substantial overlap, we decided for LPPs to analyze mean amplitudes in the time intervals 270–370 ms (P300) and 380–480 ms (P400) for electrodes P3, Pz, and P4. P100 and N170 peaks were detected bilaterally at electrodes O1 and O2 (50–100 ms) and electrodes P7 and P8 (100–200 ms), respectively.

### Statistical Analysis

For data analysis, linear mixed effects models [60] were implemented using SAS 9.1 (SAS Institute Inc.). Linear mixed models are particularly well suited for repeated measurement designs within the same individual that can lead to positive correlations between measurements. In all analyses of variance (ANOVAs), Subjects served as a random effect [61], whereas all other factors were fixed effects. Significant effects in an ANOVA were further analyzed by calculating relevant contrasts, where rejection of the null hypothesis was controlled by Holm's sequential rejection algorithm [62]. Original *p*-values that remained significant after α-correction are reported below. As a measure of effect size, partial eta squared (η_p_^2^) is reported.

In the reaction time analysis, trials were excluded in which no reaction-time button-press occurred, the answer was wrong, or the response time was below 200 ms. A 3 × 2 × 2 ANOVA with between factor Group and repeated measures factors Task (identify color, identify object) and Object (spider, flower) was performed.

Mean amplitudes in the time intervals 270–370 ms (P300) and 380–480 ms (P400) were analyzed by means of a 3 × 2 × 2 × 3 ANOVA with between factor Group and repeated measures factors Task, Object, and Laterality (left, central, right). P100 and N170 amplitudes were also analyzed with a 3 × 2 × 2 × 2 ANOVA with between factor Group and repeated measures factors Task, Object, and Laterality (left, right). Three individuals (two controls, one social phobic) had to be excluded from the analysis of LPPs due to severe occipito-parietal alpha activity. For the analysis of early visual components, three individuals (one social phobic, two spider phobics) were excluded because they showed no detectable P100 and N170 components.

## Results

Table 3 shows an overview of significant statistical results.

**Table 3:** Overview of statistical results

	*F *value	*p *value
Reaction times		
Group	F(2,53) = 3.67	*p *= .03
Task	*F*(1,53) = 13.52	*p *= .0006
Object	*F*(1,53) = 9.36	*p *= .004
Task × Object	*F*(1,53) = 15.02	*p *= .0003
P100		
Group	*F*(2,50) = 3.32	*p *= .04
Laterality	*F*(1,52) = 4.39	*p *= .04
Object × Laterality	*F*(1,208) = 4.75	*p *= .03
N170		
Object	*F*(1,50) = 16.14	*p *= .0002
Task × Object	*F*(1,208) = 5.09	*p *= .03
P300		
Task	*F*(1,50) = 19.50	*p *< .0001
Object	*F*(1,50) = 5.91	*p *= .02
Task × Object	*F*(1,364) = 22.62	*p *< .0001
Group × Task × Object	*F*(4,364) = 7.62	*p *< .0001
Laterality	*F*(2,104) = 16.91	*p *< .0001
P400		
Object	*F*(1,50) = 10.06	*p *= .003
Task × Object	*F*(1,364) = 8.33	*p *= .004
Group × Task × Object	*F*(4,364) = 5.42	*p *= .0003
Laterality	*F*(2,104) = 20.87	*p <*.0001

### Reaction Times

Participants failed to respond in 1.1% of all trials, and incorrect responses were observed in 1.2% of all trials. Groups did not differ in total mistakes (omissions & incorrect choices), Kruskal-Wallis χ^2^(2, N = 56) = 1.7, *p *= .43.

Participants identified the color of an object faster than the object itself, main effect of Task, *F*(1,53) = 13.52, *p *= .0006, η_p_^2 ^= .20. Furthermore, a main effect of Object, *F*(1,53) = 9.36, *p *= .004, η_p_^2 ^= .15, and an interaction of Task × Object, *F*(1,53) = 15.02, *p *= .0003, η_p_^2 ^= .22, were observed. All groups showed similar color identification latencies for schematic spiders and flowers. Thus, spider phobic individuals showed no emotional Stroop interference when identifying the color of schematic spiders (see Table 4). However, in the object identification task, all groups identified schematic spiders faster than schematic flowers, *p *< .0001.

**Table 4:** Reaction times

Condition	Control Group		Spider Phobics		Social Phobics	
	
	*M*	*SD*	*M*	*SD*	*M*	*SD*
Color identification						
Spider	522.77	100.45	479.40	74.20	501.24	75.51
Flower	523.13	99.64	478.11	69.81	501.69	72.08
Object identification						
Spider	540.62	72.72	469.99	76.43	541.51	54.00
Flower	563.93	64.25	501.84	55.72	561.00	56.92

Mean response latencies (*M*) and standard deviances (*SD*) for the color and object identification of schematic spiders and flowers for each group.

Furthermore, a main effect of Group, *F*(2,53) = 3.67, *p *= .03, η_p_^2 ^= .12, was observed. Subsequent contrasts showed that individuals with spider phobia responded faster than controls and social phobic persons, *p *= .01.

### Event-Related Potentials

#### Analysis of P100 amplitude

**Figure 2:**
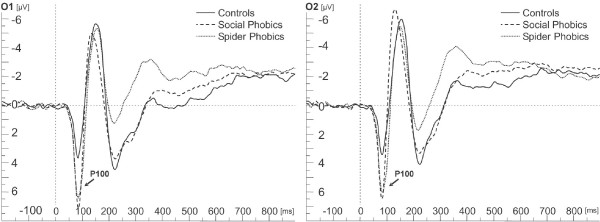
**Occipital event-related potentials**. Grand average ERPs on electrodes O1 (left) and O2 (right) for individuals with social phobia, individuals with spider phobia and control persons.

ANOVA revealed a main effect of Group, *F*(2,50) = 3.32, *p *= .04, η_p_^2 ^= .12. Larger P100 amplitudes for social and spider phobic persons were observed compared to controls, *p *= .01 (see Figure 2). P100 amplitudes were larger over left compared to right sites, main effect of Laterality, *F*(1,52) = 4.39, *p *= .04, η_p_^2 ^= .09. However, as the interaction Object × Laterality revealed, *F*(1,208) = 4.75, *p *= .03, η_p_^2 ^= .10, this difference was only present for schematic flowers, *p *= .007, and not for schematic spiders.

#### Analysis of N170 amplitude

**Figure 3:**
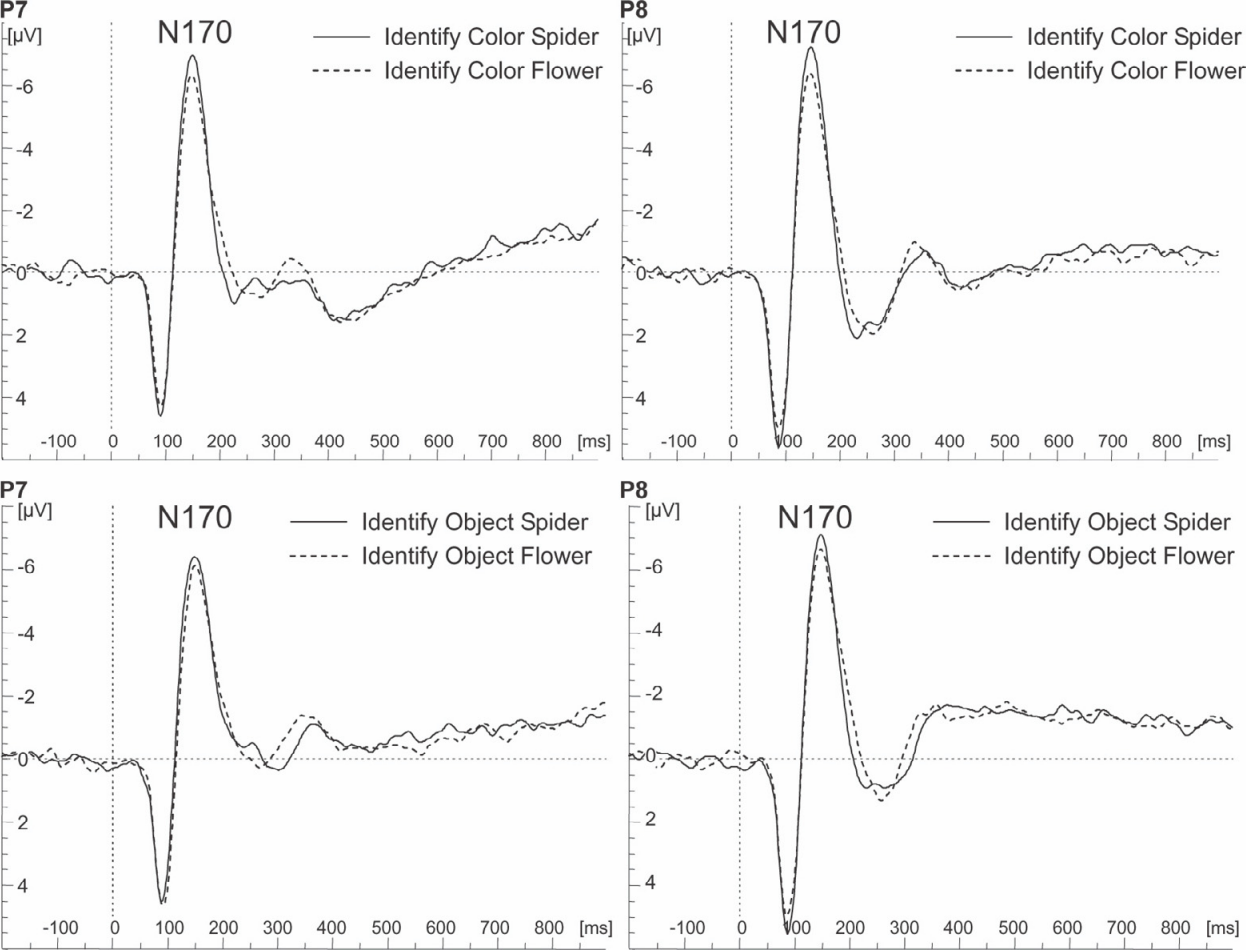
**Occipito-temporal event-related potentials**. Grand average ERPs on electrodes P7 and P8 for color (upper row) and object (lower row) identification of schematic spiders and flowers.

Larger N170 amplitudes for spiders than flowers were observed, main effect of Object, *F*(1,50) = 16.14, *p *= .0002, η_p_^2 ^= .24 (see Figure 3). This effect was modulated by task, interaction Task × Object, *F*(1,208) = 5.09, *p *= .03, η_p_^2 ^= .08. In both tasks, spiders led to larger N170 amplitudes than flowers. However, this effect was more pronounced in the color identification task, *p *< .0001, than in the object identification task, *p *= .04. No significant main effect of or interaction with group was observed.

#### Analysis of P300 amplitude

**Figure 4:**
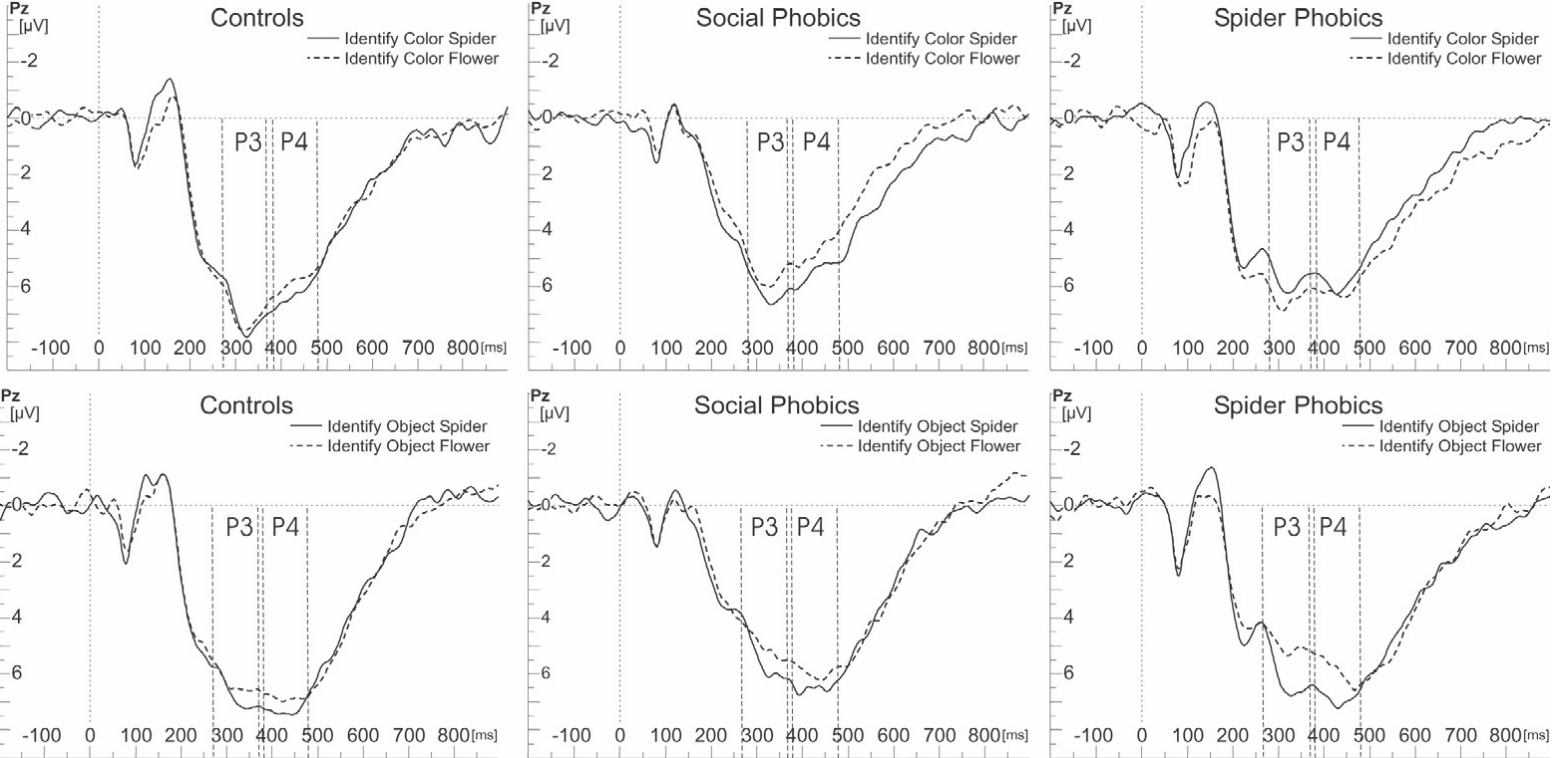
**Parietal event-related potentials**. Grand average ERPs on electrode Pz for color (upper row) and object (lower row) identification of schematic spiders and flowers for each group.

Color identification led to larger P300 amplitudes than object identification, main effect of Task, *F*(1,50) = 19.5, *p *< .0001, η_p_^2 ^= .28. Spiders led to larger P300 amplitudes than flowers, main effect of Object, *F*(1,50) = 5.91, *p *= .02, η_p_^2 ^= .11. However, this effect was only present in the object, but not in the color identification task, interaction of Task × Object, *F*(1,364) = 22.62, *p *< .0001, η_p_^2 ^= .11. Furthermore, this effect was only significant in the spider phobic group, as the interaction of Group × Task × Object, *F*(4,364) = 7.62, *p *< .0001, η_p_^2 ^= .14, revealed, although it was present as a tendency also in the control groups (see Figure 4). Subsequent contrasts confirmed larger P300 amplitudes in spider phobic persons in response to schematic spiders than flowers in the object identification task, *p *= .005. In addition, there was a tendency towards smaller P300 amplitudes in spider phobic persons when identifying the color of a spider which, however, was not significant.

A main effect of laterality, *F*(2,104) = 16.91, *p *< .0001, η_p_^2 ^= .25, showed larger P300 amplitudes over central compared to left or right sites, both *p *< .0001, as well as over right compared to left sites, *p *= .002.

#### Analysis of P400 amplitude

Larger P400 amplitudes were observed in response to schematic spiders than flowers, main effect of Object, *F*(1,50) = 10.06, *p *= .003, η_p_^2 ^= .17. However, this effect was only present in the object identification task but not in the color identification task, interaction of Task × Object, *F*(1,364) = 8.33, *p *= .004, η_p_^2 ^= .05. Although this effect was as a tendency present in all groups, it was most pronounced in individuals with spider phobia (compare Figure 4), interaction of Group × Task × Object, *F*(4,364) = 5.42, *p *= .0003, η_p_^2 ^= .11. Subsequent contrasts confirmed that schematic spiders led to larger P400 amplitudes in individuals with spider phobia than schematic flowers when the task was to identify the object, *p *< .0001, whereas in the control groups the effect was not significant after α-adjustment. In the color identification task spider phobic individuals again showed a tendency towards smaller P400 amplitudes in response to schematic spiders than flowers, which, however, was not significant.

P400 amplitudes were maximal at central electrodes, main effect of Laterality, *F*(2,104) = 20.87, *p *< .0001, η_p_^2 ^= .29. Subsequent contrasts revealed larger P400 amplitudes at central sites compared to left, *p *< .0001, and right hemispheric sites, *p *< .0001.

## Discussion

The analysis of behavioral data showed that persons with spider phobia responded faster than both control groups when identifying schematic spiders in the object identification task. However, it must be noted that individuals with spider phobia responded generally faster than the control groups, while all groups identified schematic spiders faster than flowers. In contrast to the original hypotheses, no emotional Stroop interference was observed in spider phobic individuals when identifying the color of schematic spiders.

Analysis of ERPs revealed that both social phobic and spider phobic individuals showed larger P100 amplitudes compared to non-phobic controls. Furthermore, all groups, whether spider phobic or not, showed larger N170 amplitudes in response to schematic spiders than flowers. Finally, individuals with spider phobia showed enhanced LPPs (P300 and P400), in response to spiders compared to flowers in the object identification task.

### Behavioral Data

#### Facilitation for schematic spiders

All groups identified schematic spiders faster than schematic flowers, supporting Öhman's theory [1] that spiders are fear-relevant (ancestral) stimuli which are processed with high selectivity and priority by all human beings, whether phobic or not. However, in a similar study design with "real" spider pictures, Kolassa et al. [18] found no facilitated responses for spiders in non-spider-fearful individuals. Thus, the question arises why spider-non-fearful persons show facilitated identification of schematic but not photographic spider images. It is possible that the cause for the different findings lies in the experimental design of the studies. On the one hand, the task performance may have been easier when using schematic as compared to veridical pictures, and on the other hand, whereas the present study used only two types of stimuli (schematic spiders and flowers), Kolassa et al. [18] used three types of trial-unique stimuli (photographic spider, bird, and flower images). These differences in task difficulty are also reflected in the smaller standard deviations in the present compared to the Kolassa et al. [18] study. Because data were less noisy it is possible that even small effects reached significance in the present study.

#### Hypervigilance in spider phobic individuals

Persons with spider phobia responded generally faster than both control groups, presumably due to a general "behavioral" hypervigilance in phobic persons. According to Beck et al. [[63], p. 31], "The [anxious] patient is hypervigilant, constantly scanning the environment for signs of impending disaster or personal harm." According to Eysenck [64-66], there are two ways in which individuals high in trait anxiety show hypervigilance: *general *hypervigilance or distractability is demonstrated by a propensity to attend to any task-irrelevant stimuli presented, and *specific *hypervigilance is demonstrated by a tendency to attend selectively to threat-related rather than neutral stimuli. But why should such a general behavioral hypervigilance be present in spider phobic but not in social phobic persons? One explanation for the lack of similarly faster responses of social phobic persons might be the larger depression and general anxiety-related psychopathology in this patient group, which reciprocally diminishes fear reactivity. Increasing psychopathology along the anxiety disorder spectrum (specific phobia → social phobia → panic disorder with agoraphobia → generalized anxiety disorder) is a well-established finding [67-69]. Lang et al. [69] showed that individuals suffering from specific phobia are most reactive to specific cues in the environment, e.g., their startle reflex was most pronounced in a startle probe modulation task involving imagery of social and survival threat. However, defensive reflexes were diminished with increasing generalized anxiety and depression. It has been shown that this reflex pattern is not specific to fear cues that are related to phobics' clinical problems [68,70]. Lang et al. [69] suggested that generalized anxiety and comorbid depression additively attenuate startle potentiation to imagined threat in anxiety disordered patients.

#### Absence of Stroop interference

This study found no emotional Stroop interference in spider phobic persons when identifying the color of schematic spiders, which is consistent with a preceding study by Kolassa et al. [18] that also found no emotional interference in a similar experimental paradigm with spider phobic individuals. As discussed by Kolassa et al. [18], several explanations might account for this finding: a two-choice color identification task might be too simple for interference to occur, or else emotional interference might be smaller or even absent when using a manual response mode instead of a vocal one (compare [71,72]). Furthermore, different formats of the emotional Stroop task might not be psychometrically equivalent (card vs. computer, blocked vs. randomized designs; compare [73-77]. Finally, whether pictorial or linguistic stimuli are used seems to affect the magnitude of emotional Stroop interference: No study so far reported larger interference for pictorial than for linguistic stimuli [13,78]. Because pictures of spiders seem to be more ecologically valid stimuli for spider phobic persons than spider-related words, one would have expected the opposite effect. Possibly, linguistic stimuli increase task difficulty, thus enhancing interference.

### Early Visual Components: P100 and N170

Phobic persons displayed larger P100 amplitudes than non-phobic individuals regardless of the processed stimulus material or task, in accordance with a reanalysis of our previous study [18] in which originally P100 and N170 amplitudes were not analyzed.

Because the P100 is thought to be attention-sensitive (for a review see [12,37]), the present data may be indicative of a "cortical" hypervigilance in phobic individuals [64-66] and stronger attentional processing of incoming stimuli in phobic than in non-phobic persons. Both phobic groups were in situations they fear: persons with social phobia fear performance situations such as the experimental setting, while spider phobic persons viewed pictures of their feared object.

The processing of schematic spiders elicited larger N170 amplitudes than the processing of schematic flowers. This also is in accordance with the reanalysis of our previous study [18]: spiders led to largest N170 amplitudes, flowers to lowest with birds in between and with all contrasts highly significant. So far, the literature on the N170 component mainly focuses on face processing mechanisms (e.g., [38,39]). More recently, it has been proposed that the amplitude of the N170 depends on the perceptual expertise in discriminating objects [40-42]. Thus, the enhanced N170 for spiders in this study might be interpreted on the one hand as being due to a higher level of expertise in processing schematic spiders as compared to the more artificially looking flower stimuli, on the other hand it might reflect a general advantage for fear-relevant compared to neutral stimuli. Nevertheless, it remains intriguing that spider phobic persons do not exhibit an additional expert effect on the N170 over and beyond the control groups, as found both in the present study as well as in the reanalysis of the data of our previous study. Although the debate cannot be resolved at this point, a new interpretation of the N170 in addition to face processing and expertise effects might be called for.

### Parietal Late Positive Potentials (LPPs)

The larger P300 and P400 amplitudes in response to schematic spiders compared to schematic flowers in the spider phobic group when the task was to identify the object fit the well-documented finding that parietal LPPs are influenced by the emotionality of presented stimuli (e.g. [24,27,28,35]). Furthermore, the findings are in accordance with previous studies that found larger parietal LPPs in spider phobic persons when viewing veridical spider pictures [18,23]. Thus, the data suggest that schematic spiders trigger meaning-related evaluative processes in the brains of spider phobic persons similar to those observed when processing real spider pictures.

Whereas enhanced LPPs in response to spiders were present in both tasks in spider phobic individuals in our previous study [18,23], in the present study such an effect was only present in the object identification task, i.e. when attention was directed explicitly at the spider stimulus instead of at its color. Indeed, there was even a tendency towards smaller amplitudes in response to schematic spiders than flowers in persons with spider phobia in the color identification task. The present results might suggest that spider phobics try to avoid detailed processing of spider pictures if the task does not require object processing, and that it might be easier to avoid processing of schematic spiders than real spiders when only their color has to be identified, by processing low-level visual elements of schematic pictures, conceptually remaining on the level of a basic arrangement of lines. On the other hand, photographic stimuli as used in our previous study may not be so easily reducible to abstract basic elements, engaging resources in processing the stimulus category even in color identification and thus showing the effects of differential processing of phobic stimuli by spider phobics.

## Conclusion

Individuals with spider phobia showed larger LPPs when their task was to identify schematic spiders, suggesting that these stimuli prompted phobia-specific responses in these individuals. Results indicate that schematic spiders are sufficient to evoke differential processing in spider-fearful compared to non-spider-fearful persons. Furthermore, all groups, whether spider phobic or not, exhibited differential behavioral and ERP responses in response to schematic spiders compared to schematic flowers, suggesting that spider stimuli are special stimuli all individuals, whether phobic or not. Finally, consistent with current models on hypervigilance in the anxiety disorder spectrum, this study found behavioral and electrophysiological evidence for an enhanced hypervigilance in individuals with spider phobia.

## Competing interests

The author(s) declare that they have no competing interests.

## Authors' contributions

ITK designed the study, acquired the data, performed the data analysis and statistics, and wrote the manuscript. FM developed the stimuli for the study and participated in conceiving the study. SK programmed the paradigm and assisted in statistical analysis and writing of the manuscript. WHRM conceived the study, acquired funding and provided infrastructure. All authors read and approved the final manuscript.

## Pre-publication history

The pre-publication history for this paper can be accessed here:


